# Cortisol advantage of neighbouring the opposite sex *in utero*

**DOI:** 10.1098/rsos.171636

**Published:** 2018-09-05

**Authors:** R. Fishman, Y. Vortman, U. Shanas, L. Koren

**Affiliations:** 1 The Mina and Everard Goodman Faculty of Life Sciences, Bar-Ilan University, Ramat-Gan 5290002, Israel; 2 Hula Research Center, Department of Animal Sciences, Tel-Hai College, Upper Galilee 1220800, Israel; 3 Faculty of Life Sciences, University of Haifa—Oranim, Tivon 3600600, Israel

**Keywords:** glucocorticoids, sex ratio, hair testing, intrauterine position

## Abstract

Population sex ratios naturally fluctuate around equality. It is argued that the production of an equal number of male and female offspring by individual parents should be favoured by selection, if all costs and benefits are equal. Theoretically, an even sex ratio should yield the highest probability for a fetus to be adjacent to a fetus of the opposite sex *in utero*. This may cause developmental costs or benefits that have been overlooked. We examined the physiological and developmental parameters associated with *in utero* sex ratios in the nutria (*Myocastor coypus*), an invasive wildlife species with a strong reproductive output. Using hair testing, we found that litters with even sex ratios had the highest average cortisol levels. Fetuses neighbouring the opposite sex exhibited longer trunks than those neighbouring the same sex, which might imply better lung development. Our results are the first, to our knowledge, to link intra-utero sex ratios and fetal cortisol and suggest that fetal cortisol might be a mechanism by which even sex ratios are maintained via developmental advantages.

## 1. Introduction

Evolutionary forces and developmental constraints shape prenatal sex ratios [1,2]. Even sex ratios are connected with negative frequency-dependent selection in most species as well as individuals [3–5]. However, other evolutionary forces favour skewed offspring sex ratios as a result of fitness benefits [6,7]. Sex allocation allows the parents to invest in the more reproductively successful sex in accordance with their condition [7], to respond to changes in available resources [6,8] or social status [9], and to enable trade-offs between size, number and sex of the offspring [10]. While ultimate causes may favour skewed or even sex ratios, within utero mechanistic costs and benefits may also shape the adaptive value of different sex ratios. For instance, if the offspring's fitness is dependent on litter sex composition, selection may favour maternal adjustment of the sex ratio [11]. The *in utero* sex ratio affects the probability of a fetus to neighbour opposite-sex fetuses. In humans, studies on twins show increased survival for fetuses and neonates of opposite-sex twins compared with same-sex twins, as well as dizygotic twins [12–18]. However, in some species, selection favours same-sex litters [11].

Maternal glucocorticoids are often presented in the literature as indicators of maternal stress (e.g. [10,19,20]). In late pregnancy, however, glucocorticoids serve a crucial role in preparing the fetus for the extrauterine environment, and their elevation has been documented in many species (e.g. [21–23]). Glucocorticoids such as cortisol are responsible for multiple developmental milestones, and have been shown to be associated with physiological maturity and fetal survival [22,24,25]. The transition from the protected uterine environment to the outside world poses many demands and challenges. For example, the trans-placental glucose supply must be rapidly replaced by an independent source. These immediate glucose needs, provided by the liver glycogen stores, and gluconeogenesis in the long run, are dependent on the secretion of peripartum fetal glucocorticoids (e.g. [22,26]). The neonate gastrointestinal tract must be prepared for food digestion after birth via maturation processes, in which glucocorticoids play a crucial role [22,27]. Most importantly, cortisol promotes lung maturation both structurally and functionally [22,23]. These effects have led to defining corticosteroid administration before anticipated preterm birth as ‘one of the most important antenatal therapies available to improve newborn outcomes' [28, p. e102].

While multiple maternal factors, including glucose levels (e.g. [1]), testosterone (e.g. [29]) and glucocorticoids (e.g. [10,19,20]), have been considered in shaping prenatal sex ratios (see [30] for a recent synthesis), here we wish to examine how the sex ratio might morphologically and hormonally affect the fetal condition *in utero*. Specifically, we investigated whether litter sex ratios explained the levels of circulating cortisol in litters and individual fetuses, and whether the condition of the individual fetus was influenced by the sex of its neighbour. Based on sexual asymmetry in competitive ability [31], we hypothesized that fetuses in competitive sex-biased litters would have higher cortisol levels owing to competition for resources [32]. Using the feral nutria (*Myocastor coypus*), where sexual asymmetry is pronounced as male offspring spend more time suckling from the highest yielding teat and grow faster than females [33], we examined the influence of sex ratios on the uterine environment and fetal interactions. We predicted that both male and female fetuses in male-biased litters will have higher cortisol levels.

## 2. Methods

### 2.1. Sample collection

Culled animals were collected at the Agamon Hula Park. A total of 153 females were collected, of which 117 (76%) were pregnant. Our sample for this study was composed of 82 pregnant nutrias and their 461 fetuses at a pregnancy stage of 52–138 days. The average litter size was 5.6 fetuses. Twenty-two females whose pregnancy stage was 106–138 days had fetuses with sufficient hair to allow hair testing for steroid quantification (overall 121 fetuses: 57 males and 64 females). The length from shoulders to base of tail could be accurately measured in 24 litters containing 127 fetuses aged 105–138 days of pregnancy. Total body length was measured in 47 litters containing 246 fetuses, whose pregnancy stage was 77–138 days, and fetal weight was attained for all 82 litters.

Estimation of pregnancy stage followed Newson's formula: estimated age = 43.69 + 14.27*^3^√Fetal weight (1966) [34], cross-validated with multiple fetal morphometric measurements presented by Felipe & Masson [35] and Sone *et al*. [36]. Pregnant females were weighed using a spring scale (Pesola, Switzerland, 10 kg capacity, 100 g accuracy). Fetuses were weighed using an analytic balance to the nearest 0.01 mg (Precisa, Switzerland). Morphometric measurements (total length, length from nose to tail base, length from shoulders to tail base and crown-to-rump length) were conducted using a measuring tape to the nearest millimetre. One of the specimens was not intact and, thus, could not be fully measured.

### 2.2. Offspring sex ratio and intrauterine position

Fetuses were sexed based on internal and external morphology, validated using molecular tools. For external sexing, we used the anogenital distance (AGD), an accepted proxy for early androgen exposure based on testosterone's responsibility for perineal tissue elongation [37]. We validated AGD by internal examination of 10 male fetuses and 12 female fetuses. The AGD index was calculated by dividing fetal AGD length by fetal weight [38]. We found that male nutria fetuses had significantly longer AGDs (AGD index: *t*_241_ = 14.8; *p* < 0.001). In addition, for sexing fetuses that were less than 11 weeks old, we used published primers for the *Sry* gene [39], which is only expressed in males. This method was validated using four adult males, four adult females, two male fetuses and three female fetuses whose internal and external genitalia were examined. We used the housekeeping gene 12S as a positive control, and we used an adult female as a negative control. Upon dissection, the intrauterine position for each fetus was noted by the uterus horn (left or right), and its location relative to the ovary (the closest fetus was denoted as number 1). Fetuses that had a neighbouring fetus of the opposite sex on either or both sides were termed P1 (i.e. proximity to the opposite sex), while fetuses that were only next to fetuses of their own sex were termed P0.

### 2.3. Cortisol measurement

We measured cortisol, the main glucocorticoid produced by the nutria adrenal glands [40,41], in hair samples of 22 wild female nutrias and their 121 fetuses. Hair steroids reflect integrated long-term circulating levels [42]. Thus, fetal hair provides a window to intrauterine processes. Hair cortisol was extracted and quantified using our published protocol [43,44].

Briefly, the hair was shaved and washed to remove external contaminants. Cortisol was quantified using commercial ELISA kits (Salimetrics Europe, Newmarket, UK). The manufacturer reported antibody cross-reactivity of 19.2% with dexamethasone, and less than 0.568% with all other steroids. Kits were validated for nutria hair by showing linearity (0.5–10 mg hair) and parallelism between serially diluted hair extracts (representing 0.5–10 mg) and kit standards (slope covariance *p* = 0.36). The intra-assay coefficient of variation (CV) was 6.61% for six repeats on the same plate, and the inter-assay CV was 8.17% across eight plates. Recovery was calculated to be 90.93% by spiking hair samples with a known cortisol concentration.

### 2.4. Statistical analysis

Sex ratios were normally distributed (Shapiro–Wilk *W* test, *W* = 0.98; *p* = 0.152). Hair cortisol levels were transformed to achieve normal distribution (via Johnson SI transformation for litters and Johnson Su transformation for individual fetuses and maternal cortisol). Cortisol was related to estimated pregnancy stage both in individual fetuses (model *R^2^* = 0.69; *F*_1,18_ = 7.18; *p* = 0.0155) and in litter averages (*R^2^* = 0.21; *F*_1,20_ = 5.31; *p* = 0.032). Therefore, it was included in all models. In all tests that included individual fetuses, maternal identity was included as a random factor to account for the fetal uterine environment. Nonlinear (second-order polynomial) regression was fitted to the associations between sex ratios and cortisol, following visual inspection. A generalized linear model with a Poisson distribution was used to explain the number of fetuses adjacent to an opposite-sex fetus, where sex ratio (as a polynomial to a second degree) and litter size were the model effects. The number of fetuses adjacent to an opposite-sex fetus in a litter was corrected for litter size (*R^2^* = 0.34; *F*_1,74_ = 37.57; *p* < 0.0001), and the residuals were used to predict the average litter cortisol in a linear model. We used linear mixed models to predict fetal length, with pregnancy stage, sex and proximity to opposite-sex fetuses as model effects, and again with uterine horn location. Fetal weights did not distribute normally owing to a higher representation of fetuses from an early pregnancy stage, because these litters are naturally larger. Dispersion (the ratio of deviance to degrees of freedom) was accounted for in the model using a Possion distribution. We tested whether maternal cortisol levels were related to individual and average litter fetal cortisol levels using linear mixed models that included pregnancy stage and litter size. Model fitting was done in JMP (v. 12, SAS Inc.).

## 3. Results

**Figure 1.**
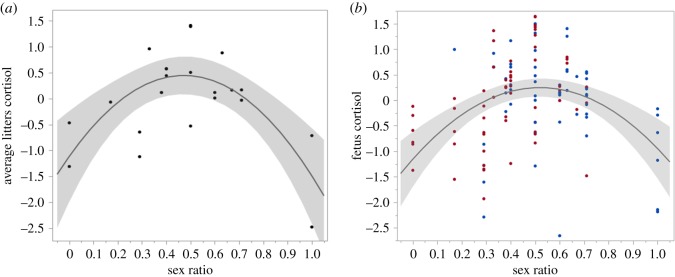
Association between sex ratio in the last trimester and cortisol levels in nutria fetuses. Sex ratios vary between 0 (all-female litter) and 1 (all males). Fetal cortisol was transformed using the Johnson SI (for litter averages) and Johnson Su (for fetuses) transformations, in a model including pregnancy stage and litter size. Distribution of sex ratios is normal, average sex ratio is 0.48, which is not significantly different than even sex ratios (i.e. 0.5). (*a*) Average litter cortisol levels; (*b*) individual fetus cortisol levels (males in blue, females in red). Grey area indicates 95% confidence intervals of the second-degree polynomial fit.

No sex differences were found in fetal hair cortisol levels (*F*_1,102_ = 0.26, *p* = 0.61). We found that litter average cortisol levels could be predicted using pregnancy stage, sex ratio and litter size (whole model *F*_4,17_ = 7.895; *p* = 0.0009). However, only estimated pregnancy stage and sex ratio were significant (table 1), and the highest cortisol levels were seen at even sex ratios (figure 1*a*). A similar relationship was seen in individual fetuses, in a model that included sex ratio, pregnancy stage and litter size (table 2 and figure 1*b*), while taking into account maternal effect (restricted maximum likelihood (REML) random effect; variance ratio 0.95; Wald *p* = 0.014). We did not find a relationship between maternal cortisol and individual fetal or average litter cortisol levels.

**Table 1.** Whole model of association between estimated pregnancy stage, sex ratio and litter size as predictors of litter average cortisol levels. (Italics indicate statistically significant values.)

parameter	estimates	s.e.	*F* ratio	prob *>* *p*
estimated pregnancy stage	0.05	0.02	*8*.*65*	*0.0091*
sex ratio^2^	−5.43	2.21	6.05	*0.025*
sex ratio	−0.12	0.57	0.04	0.84
litter size	0.12	0.11	1.11	0.31

**Table 2.** Whole model of association between estimated pregnancy stage, sex ratio and litter size as predictors of individual fetus cortisol levels. (Italics indicate statistically significant values.)

parameter	estimates	s.e.	*F* ratio	prob *>* *p*
estimated pregnancy stage	0.048	0.018	7.02	*0.017*
sex ratio^2^	−4.88	2.15	5.14	*0.036*
sex ratio	0.18	0.60	0.09	0.77
litter size	0.08	0.12	0.47	0.5

We found that nutria fetuses neighbouring an opposite-sex fetus *in utero* are longer from shoulder to base of tail, regardless of sex (*F*_1,112_ = 8.47, *p* = 0.0044; table 3). There were no differences in other length measurements, which were expected to intercorrelate with this measurement (total length *p* = 0.11; length from nose to tail base *p* = 0.36; length from crown to rump *p* = 0.48). However, we found a significant maternal effect on fetal length (REML random effect; variance ratio 1.015; Wald *p* = 0.012), which is expected considering the relationships between maternal and offspring length [45,46]. We further found that both litter size and sex ratio influenced the prevalence of opposite-sex neighbours (whole model *χ*_3_^2^ = 117.94, *p* < 0.0001; sex ratio *χ*^2^ = 8.74, *p* = 0.003; sex ratio^2^ *χ*^2^ = 50.65, *p* < 0.0001; litter size *χ*^2^ = 49.2, *p* < 0.0001). As statistically expected, larger litters contained more fetuses with opposite-sex neighbours, and the more even the sex ratio, the more fetuses had opposite-sex neighbours. We also found that litters with a higher number of fetuses neighbouring an opposite-sex fetus had higher levels of average cortisol, after correcting for litter size (*R^2^* = 0.25; *F*_1,17_ = 5.77; *p* = 0.028).

**Table 3.** Whole model of association between proximity to opposite-sex fetus, estimated pregnancy stage, sex, the interaction between sex and proximity to opposite-sex fetus as predictors to fetal length from shoulder to base of tail. (Italics indicate statistically significant values.)

parameter	estimates	s.e.	*F* ratio	prob *>* *p*
estimated pregnancy stage	0.15	0.014	117.25	*<0.0001*
proximity to opposite-sex fetus	−0.189	0.065	8.47	*0.0044*
sex	−0.073	0.065	1.27	0.26
sex × proximity to opposite-sex fetus	−0.076	0.064	1.43	0.23

Including nutria fetuses of all stages, we found that individuals located at the end of the uterus horn tended to be longer (*F*_1,207_ = 6.13, *p* = 0.014) and heavier (*F*_1,366_ = 7.75, *p* = 0.006), regardless of sex, which might suggest a crowding effect [47]. In this model, both pregnancy stage and maternal identity, as expected, influenced fetal length and weight (REML random effect; variance ratio 0.195, Wald *p* = 0.0406 for length, and variance ratio 8.75, Wald *p* < 0.0001 for weight). However, there were no differences in hair cortisol between fetuses positioned in the middle of the uterus horn and fetuses at the end of the uterus horn.

## 4. Discussion

The main finding of this study is that litter sex ratios, in a model with pregnancy stage, and litter size explain fetal cortisol levels. Surprisingly, we found that cortisol varied in a parabolic manner, so that litters with even sex ratios showed the highest average cortisol, suggesting a previously unexplored adaptive advantage to even sex ratios in litter-bearing mammals. Cortisol was not a sign of stress or crowding in our study, for fetuses located in the middle of uteral horns tended to be smaller, but not with higher cortisol. We infer that high glucocorticoid levels in even-sexed litters in our study might indicate optimal fetal development, as well as stage-appropriate hypothalamic–pituitary–adrenal (HPA) axis activation. It is well documented that cortisol promotes fetal system maturation (e.g. [22–24,26,27,48,49]). Its elevation in late pregnancy is especially profound in precocial species [22], and could be related to the critical role of glucocorticoids in preparing the fetus for survival outside the uterus (e.g. lung, kidney and small gut maturation, initiation of glycogen in the liver) [22]. We found that skewed sex ratios, males or females, were associated with lower cortisol. Hypocortisolism may be a result of chronic maternal stress, which might serve to prevent preterm delivery [50]. Perhaps in mammals, cortisol is also involved in maintaining even sex ratios through stabilizing selection, where its ‘protective’ effect enhances development in mixed-sex litters, though at this stage, we cannot disentangle cause and effect. Possibly, more even litter sex ratios and well-mixed intrauterine positions result in a different hormonal profile than when there is a biased litter sex ratio, and the former profile may be more beneficial to development than the latter. Under such conditions, more ‘optimal’ patterns of cortisol expression are more likely to be found in the context of even litter sex ratios.

Analysis of individual fetuses, in addition to litters, reinforced our results. We found that from a fetal perspective, being next to a fetus of the opposite sex seems to be advantageous. Fetuses neighbouring an opposite-sex fetus *in utero* were longer from shoulder to base of tail, while not different in any other length measurement. Human trunk length is measured from the top of the thorax to the bottom of the urinary bladder [51], which is analogous to our measurement of fetal length. A longer trunk might imply better lung development, a prerequisite for suitable fetal development [52]. In humans, trunk length is associated with lung volume [53], while in neonates, body length is associated with lung functional residual capacity, which is responsible for adequate gas exchange [54]. Synthetic glucocorticoids (e.g. dexamethasone) in preterm infants help prevent and treat lung disease through promoting lung, surfactant and antioxidant enzyme system maturation [48,49], while increasing lung volume [55]. Though life-saving, an excess in glucocorticoids via maternal stress or preterm treatment of synthetic glucocorticoids might also have adverse consequences, such as lower birth weights, and in adulthood, an increased risk for higher blood pressure and cardio-metabolic and behavioural disorders (e.g. [56–58]). In humans, findings from studies on twins suggest that opposite-sex twins have an advantage over same-sex twins, also dizygotic twins, in terms of survival [12–18] and birth weight [12,59]. For example, mortality rates are greatly increased for twins whose co-twin died in the neonatal period in the case of same-sex twins, but not opposite-sex twins [12]. Other advantages to opposite-sex twins in humans are heavier birth weight [12,59], at least for males [60], and longer gestation [61], perhaps owing to the tendency of female fetuses to have longer gestations [61]. In cotton-top tamarins (*Saguinus oedipus*), male twins had significantly lower survival rates than those born in male–female litters [62]. However, in other systems, opposite-sex co-twins can have adverse effects. For example, in Soay sheep, female lambs with a male co-twin were shown to have reduced birth weight and lower lifetime breeding success relative to those with a female co-twin [11]. In some rodents, females located between two males *in utero* show masculinized anatomical, physiological and behavioural features (reviewed in [63]). While many studies looked at the effects of testosterone transport from male to female fetuses [63], we could not find similar studies on the transport of fetal glucocorticoids *in utero*. We also do not know of studies that measured fetal cortisol in same- and opposite-sex twins.

Previously we found that in nutrias litter size did not influence sex ratios, and that heavier females had male-biased sex ratios [64]. We also found that male fetuses in the last stages of pregnancy were heavier and longer than female fetuses [64]. It is possible that a successful strategy for having many male fetuses is to include an equal number of female fetuses. This would reduce intrauterine crowding, which might result from multiple male fetuses, which are larger.

Overall, our results provide a novel direction for explaining the mechanisms involved in maintaining even sex ratios. Female nutrias in good condition are known to abort small, all-female litters [65]. Sex-selective abortion and fetal resorption can be mechanisms for adjusting sex ratios, and might explain the under-representation of unisex litters in nature, contrary to binomial expectations [66]. Using necropsies of culled nutrias, we found potential benefits for even-sex litters. Our findings suggest that even-sex litters have a developmental advantage, possibly mediated by elevated glucocorticoids. Although low fetal cortisol levels (i.e. low activation of the HPA axis) can be considered a desirable condition, we suggest otherwise. Our results insinuate an overlooked advantage for high cortisol levels. This may be especially important for semi-aquatic mammals like the nutrias, which require fast and complete lung maturation for offspring to enter the water while still suckling. As our study used culled nutria, it cannot evaluate survival following birth. However, the vast amount of literature on the maturational effects of cortisol in multiple systems allows us to predict that fetuses with higher cortisol will have higher chances of survival (e.g. [22–24,26,27,48,49]). Thus, we might, on the one hand, be observing a developmental constraint on the ultimate causes driving offspring sex ratios, adding a developmental cost to skewed litters. Alternatively, cortisol might be an underlying mechanism serving the ultimate forces that maintain even sex ratios in nature.

## Data accessibility

Data available from the Dryad Digital Repository: http://dx.doi.org/10.5061/dryad.f1j384s [67].
